# On Horseback

**Published:** 1878-04

**Authors:** 


### ON HORSEBACK.

Numerous have been the devices and inventions of man, to afford his fellow man the requisite amount of exercise to keep him physically sound. So many people, now-a-days, strive to live by their wits—using their brains more than they do their hands and legs, that we have degenerated into a nation of dyspeptics, which circumstance has called forth the inventive genius of the brain-workers for the production of machinery that will afford us that exercise of muscle, without which, humanity cannot be run to its possible attainments and be free from the pains and aches, for the subjugation of which, the army of doctors is yearly employed.

These inventions have taken form in the "health-lifts," pocket gymnasiums, dumb-bells, indian clubs, and a multiplicity of other contrivances, none of which can, at all, equal a half-hour's engagement with a buck-saw, or a morning walk of a mile or so.

To make gymnastics effective, they must shape themselves toward some definite object to be attained, save the single one of securing simply and solely the exercise. When a man takes hold of the handles of a lifting machine and pulls until he is blue in the face, and then fondly imagines he has been benefiting his muscular system, he is grossly mistaken—he has not done anything of the sort. Indeed, we have seen more injury result from that sort of thing than good. Exercise, to be beneficial, should be conducted in the open air, where proper arterialization of the blood can occur through the pure oxygen taken in at the lungs, which should be correspondingly stimulated to action with that of the muscular system. Then you have immediate repair of the waste which naturally occurs from muscular activity.

Nothing so fully meets the requirements of a sluggish circulation, weak digestion and flabby and inactive muscles, as horseback riding. All the inventive talent of the brain-workers in the universe combined, are not equal to the production of a machine that will so thoroughly exercise and shake up every muscle and organ entering into the construction of physical man, as a good, square ride, for one mile, upon a trotting horse! Try it just once, and see! Start out some bright, Spring morning, when the air is pure and clear, when all nature is struggling to present her charms, and so entice you to the trial and to an observation of her budding beauties. Ride to the nearest hill-top, and when you reach the level plane beyond, venture upon a burst of speed, to try the qualities of your trotter. He will enjoy it, never fear, and cause you to bob about upon that saddle like a jumping-jack on a stick. Come in at the end of an hour, and take an inventory of your pains, aches, and sore places. If there exists a square inch anywhere upon the surface of your body, from your *glutei maximi*, up or down, that fails to bring you a report of duty well performed, as indicated by tenderness to touch; or if you do not realize that the construction of your body is wonderful, not to say peculiar, in the multiplicity of its organs, that before were undiscovered by you, then has your ride been a miserable failure, else are you not in need of manual labor or exercise.

The first time we took this prescription, we were prepared to dispute the best works upon anatomy and physiology extant, and could show to any man given to investigations of this character, some queer features in our own anatomical developement. Mounting one of our own trotting steeds, we essayed the even road of the driving park, to the end that the first attempt might be smooth, and easy of accomplishment.

We started on a brisk trot—the mare had been there before and thought that speed was what was required of her, on that particular occasion—and that little beast surpassed our wildest expectations of her capabilities in that direction. At the quarter-pole, we were convinced that our upper-jaw was constructed to close upon our under one—instead of *vice versa*—that our left lung was suspended from our shoulder blade by a slender cord that snapped a second later, and let the whole confounded thing fall into our belly. At the half-mile, our liver, stomach, and other viscera, had settled into the saddle, so that every time we sat down upon them, it did seem as though we would crowd them into our boots. At the three-quarter pole, we were conscious of chafing the saddle fearfully, while the perspiration ran in rivulets down our back, until it reached our crupper bone, where it bifurcated, and passed down each leg into our boots; while our face, in color, resembled the setting sun. Hot! Hail Columbia! a boiled lobster was no circumstance to our condition! But, as we came down that home stretch, we were amazed at the incomprehensible manner in which our legs and arms were swiveled to our body. Legs, that aforetime had done us good service upon many a trout stream, now dangled helplessly on either side of the saddle, while the liberated stirrups flailed the steed to greater efforts of speed. Arms played up and down helplessly, obeying every motion of the animal, while our spine seemed to be distorted into an anterio-posterior curvature, that brought our head on a level with our watch-pocket. At this juncture, thanks to a kind friend, the wild flight of our gallant mare was arrested, and we carefully lifted to the ground, and to this day, we cannot, for the life of us, determine how that confounded saddle touched every available spot of our body. Every muscle was tangled with its neighbor, every organ unshipped, and for the balance of the week, our meals were taken from the buffet, in silence, and standing. But, that very ride made a convert of us—we found that if exercise was what was needed in our case, we got it—lots of it, too; and, knowing what we do about that style of gymnastics, we unhesitatingly recommend the trotting-horse as the only successful exercising machine in existence, answering all the requirements of the dyspeptic.

Get up in the morning and dress for the occasion. Heavy flannels to absorb perspiration; jacket, stout pants, long boots, and a light silk cap. Take breakfast before starting— never ride with an empty stomach—then mount your horse, and take it moderately. Ride a trotter, if you would secure the full advantage of horseback exercise. After an hour, dismount, and subject yourself to a good rubbing with a crash towel, and change your flannels for fresh and dry ones. After a week, you will be able to take long excursions to the hills, and enjoy the pure, clear air and the glorious sunshine, while now and then, you can stop to admire the landscape, or pluck some wild-flowers to adorn your desk at the office. Have a companion go with you, if possible, for then is the pleasure enhanced many fold. Ride *every* morning, rain or shine, if you would have the full benefit of this prescription. So many sit upon a cushioned chair bewailing their lame and sore condition after a hard day's ride, that it becomes difficult to get them out again. The treatment must be continuous, that its efficacy may reach the maximum degree. No matter how stormy, cold, or hot it may be, or how little you feel like undertaking it, do not miss your morning ride, under any circumstances. A half hour in the saddle will put to flight all unpleasant reflections—the sore places will not be felt—and if you are suitably protected with rubber clothing, you will be surprised to see how very enjoyable even a ride in the rain is. Try it, dyspeptics—try it faithfully for three months, then write us your experience, for the benefit of others.

KIRK remarked in our hearing that he knew a father who raised and cared for ten sons, but that those ten boys could not take care of one father.

